# Efficacy and Safety of Oral NEPA Versus Fosaprepitant Plus Palonosetron for Preventing Chemotherapy-Induced Nausea and Vomiting in Patients with Nasopharyngeal Carcinoma: A Propensity-Score-Matched Retrospective Study

**DOI:** 10.3390/cancers18101533

**Published:** 2026-05-09

**Authors:** Yilin Cai, Ying Zeng, Qihang Li, Guihua Yi, Donghong Yang, Tongyuan Deng, Xiangyong Li, Haiqing Luo

**Affiliations:** 1 Department of Head and Neck Oncology, Affiliated Hospital of Guangdong Medical University, Zhanjiang 524002, China; 2 Institute of Biochemistry and Molecular Biology, Guangdong Medical University, Zhanjiang 524023, China

**Keywords:** NEPA, chemotherapy-induced nausea and vomiting, fosaprepitant, palonosetron, cisplatin, nasopharyngeal carcinoma, antiemetic

## Abstract

**Objectives**: This study compared the efficacy and safety of a single oral dose of a fixed-dose combination of netupitant and palonosetron (NEPA) with an intravenous regimen of fosaprepitant (FosAPR, 150 mg) plus palonosetron (PALO, 0.25 mg) in patients with locally advanced nasopharyngeal carcinoma (LA-NPC). **Methods**: This single-center retrospective cohort study included patients with stage III–IVa NPC who received cisplatin-based induction chemotherapy (IC) followed by concurrent chemoradiotherapy (CCRT) from January 2020 to October 2025. Propensity score matching (PSM) generated 214 patients per group. All patients also received olanzapine and dexamethasone. Complete response (CR, defined as no emesis and no rescue medication), nausea control, adverse events, and nutritional status changes were assessed across the acute, delayed, overall, and extended (0–168 h) phases. **Results**: After PSM, 214 patients were included in each group. During the first IC cycle, the oral NEPA group achieved a higher CR rate in the extended overall phase (0–168 h) than the FosAPR + PALO group (80.0% vs. 70.1%, *p* = 0.019). A similar difference was seen in the first CCRT cycle (74.8% vs. 64.5%, *p* = 0.021). The advantage persisted across subsequent cycles, with no between-group difference in the acute phase. Nausea control also favored oral NEPA: rates of no significant nausea (visual analog scale < 25 mm) during the extended overall phase were 77.1% versus 65.9% in the first IC cycle (*p*= 0.010) and 72.9% versus 60.3% in the first CCRT cycle (*p* = 0.006). Fewer patients in the NEPA group required rescue antiemetics (14.5% vs. 22.0% in the first IC cycle, *p* = 0.045), and the median time to first rescue was longer (58.3 vs. 51.3 h, *p* < 0.001). Adverse event profiles were similar between groups, with constipation being the most common. Nutritional outcomes, including weight loss ≥ 5% and severe malnutrition, did not differ significantly. **Conclusions**: For patients with LA-NPC receiving highly emetogenic chemotherapy (HEC), oral NEPA appears to offer superior and sustained chemotherapy-induced nausea and vomiting (CINV) prophylaxi with a simplified administration schedule compared with the intravenous FosAPR plus PALO regimen. These findings warrant confirmation in prospective studies.

## Simple Summary

Patients with nasopharyngeal carcinoma often receive cisplatin-based chemotherapy, which causes severe nausea and vomiting. Standard antiemetic regimens are effective but complicated to administer. This study compared a single oral capsule containing netupitant and palonosetron with a standard intravenous two-drug regimen. The oral capsule provided better protection against nausea and vomiting, especially in the days after treatment, and worked well over multiple chemotherapy cycles. Its simpler use may help more patients complete their cancer treatment without interruption.

## 1. Introduction

Nasopharyngeal carcinoma (NPC) exhibits a distinctive geographic distribution, with the highest incidence rates consistently reported in Southeast Asia, southern China, and North Africa [1,2]. Due to the anatomically concealed location of the nasopharynx, over 70% of patients present with locally advanced disease (LA-NPC). For these patients, the standard first-line treatment paradigm comprises cisplatin-based induction chemotherapy (IC) followed by concurrent chemoradiotherapy (CCRT) [1,3]. Cisplatin is unequivocally classified as highly emetogenic; in the absence of adequate prophylaxis, the risk of vomiting exceeds 90% [4]. Chemotherapy-induced nausea and vomiting (CINV) profoundly impairs patients’ functional status, compromises nutritional intake, and frequently undermines treatment adherence, all of which may negatively influence long-term survival outcomes [5,6].

The 2023 MASCC/ESMO guidelines advocate a three-drug prophylactic regimen comprising a neurokinin-1 receptor antagonist (NK_1_RA), a 5-hydroxytryptamine-3 receptor antagonist (5-HT_3_ RA), and dexamethasone for patients with head and neck cancer receiving highly emetogenic chemotherapy (HEC). The guidelines further suggest adding olanzapine for individuals deemed at high risk [7]. Despite its established efficacy, this multi-agent approach introduces considerable complexity in clinical administration. Real-world studies have consistently shown that adherence to these antiemetic guidelines in clinical practice is suboptimal, and the operational inconvenience of the treatment regimen (including medication sequence, dosage selection, drug combinations, etc.) is a major contributing factor to this poor adherence [8,9].

The standard intravenous regimen combining fosaprepitant (FosAPR) and palonosetron (PALO) carries specific limitations. FosAPR is associated with a notable risk of infusion-site reactions, reported in up to 34.7% of patients [8]. Furthermore, its physicochemical incompatibility with PALO necessitates sequential intravenous administration, further compounding clinical workload [10]. More importantly, FosAPR has a short terminal half-life of approximately 9 to 13 h [11]. This unfavorable pharmacokinetic profile may leave patients vulnerable during the delayed and extended phases of CINV, particularly beyond the first 120 h after chemotherapy. This vulnerability is clinically significant, as 10% to 27% of patients receiving guideline-directed prophylaxis continue to experience nausea up to 14–16 days post-chemotherapy, and 42% to 53% require rescue antiemetics [12,13,14]. In addition, inadequate control during the first cycle can facilitate the development of anticipatory nausea and conditioned emetic responses, thereby compromising efficacy in subsequent cycles [15].

NEPA is a fixed-dose combination antiemetic that simultaneously targets the two primary pathways mediating CINV, containing netupitant (an NK1RA) and PALO (a 5-HT3RA). Its oral, single-dose formulation offers substantial practical advantages by simplifying administration and potentially improving adherence to antiemetic guidelines [11,16]. Pharmacologically, netupitant provides prolonged NK_1_ receptor occupancy, maintaining over 90% occupancy at 96 h, and its active metabolites exhibit extended half-lives [17]. This durable receptor antagonism is expected to provide sustained protection beyond the conventional 120 h window. A phase III study of the intravenous prodrug fosnetupitant demonstrated superior complete response (CR) rates compared with FosAPR during the extended 0–168 h period after cisplatin-based chemotherapy, confirming efficacy through day seven [18]. The efficacy of oral NEPA has also been established in the moderately emetogenic chemotherapy (MEC) setting, where a randomized phase III trial showed superior CR rates compared with palonosetron alone [19]. However, evidence in the HEC setting, particularly in patients with LA-NPC receiving concurrent chemoradiotherapy, remains limited. Therefore, we conducted the present study to evaluate oral NEPA in this more challenging context.

Despite these pharmacological and practical advantages, the efficacy of oral NEPA in the specific context of LA-NPC remains unexplored. Patients with LA-NPC represent a uniquely challenging population, as they undergo multiple cycles of high-dose cisplatin during both IC and CCRT, with the latter involving concurrent radiotherapy. Radiotherapy itself can directly stimulate the dorsal vagal complex and other brainstem emetic centers, thereby increasing overall emetic risk [20,21]. Moreover, radiation-induced oral mucositis, which affects the majority of patients, can compromise oral intake and further complicate CINV management. In this demanding setting, a single-capsule antiemetic with sustained activity could offer particular value. To date, however, no study has evaluated oral NEPA across multiple cycles of combined-modality treatment in this population.

To address this evidence gap, we designed the present study to compare the efficacy and safety of oral NEPA with those of intravenous FosAPR plus PALO for CINV prevention in patients with LA-NPC receiving multi-cycle cisplatin-based IC and CCRT. Leveraging netupitant’s prolonged pharmacological activity, we specifically assessed protective durability across the extended 0–168 h period and examined whether control achieved in the first cycle influences outcomes in subsequent cycles. We hypothesized that oral NEPA would provide superior and sustained protection, particularly during the extended phase and across repeated treatment cycles, and that this durable control might attenuate the nutritional decline often exacerbated by CINV in this vulnerable population.

## 2. Materials and Methods

### 2.1. Study Design and Patients

This single-center retrospective cohort study reviewed demographic and clinical records of patients with LA-NPC (stage III-IVa, AJCC 8th edition) who completed cisplatin-based IC and CCRT at the Department of Head and Neck Oncology, Affiliated Hospital of Guangdong Medical University (Zhanjiang, China) between January 2020 and October 2025.

Inclusion criteria were as follows: male or non-pregnant female patients aged 18 to 70 years; Eastern Cooperative Oncology Group (ECOG) performance status of 0 or 1; body mass index (BMI) between 18.5 and 23.9 kg/m^2^; histologically confirmed non-keratinizing or keratinizing NPC; absence of nausea or vomiting in the week preceding each treatment cycle; no underlying conditions predisposing to nausea or vomiting (e.g., active bowel obstruction, uncontrolled intracranial hypertension, or clinically significant electrolyte imbalances requiring intervention); completion of IC and CCRT with high-dose cisplatin (≥80 mg/m^2^); adequate bone marrow function (absolute neutrophil count ≥ 1.5 × 10^9^/L, hemoglobin ≥90 g/L, platelets ≥ 100 × 10^9^/L); liver function with alanine aminotransferase and aspartate aminotransferase levels ≤1.5 times the upper limit of normal (ULN); and creatinine clearance ≥60 mL/min/1.73 m^2^.

Exclusion criteria included: receipt of other prophylactic antiemetic regimens (e.g., NK_1_RA), other highly emetogenic agents (e.g., opioids), or concomitant strong CYP3A4 inhibitors (e.g., ketoconazole, clarithromycin); missing ≥20% of key variables, including cisplatin dose, complete response status, or rescue medication records; or inability to complete the MASCC Antiemesis Tool (MAT) assessment.

### 2.2. Treatments

Both the oral NEPA group and the group receiving FosAPR combined with PALO underwent identical antitumor therapy. During IC cycles, patients received high-dose cisplatin (Jiangsu Hansoh Pharmaceutical Co., Ltd., Lianyungang, China) at 80 mg/m^2^ in combination with gemcitabine (Jiangsu Hansoh Pharmaceutical Group Co., Ltd., Lianyungang, China) at 1000 mg/m^2^ on days one and eight, repeated every three weeks for three cycles. During CCRT cycles, patients received high-dose cisplatin at 100 mg/m^2^ without other chemotherapy drugs, also every three weeks for three cycles. All patients underwent intensity-modulated radiation therapy. Target volumes were defined using computed tomography and included the gross nasopharyngeal tumor volume, gross lymph node tumor volume, high-risk clinical target volume, and low-risk clinical target volume. Prescription doses were 69.96 Gy for gross tumor volume, 60 Gy for high-risk clinical target volume, and 54 Gy for low-risk clinical target volume, delivered once daily, five days per week, for 33 fractions.

**Figure 1:**
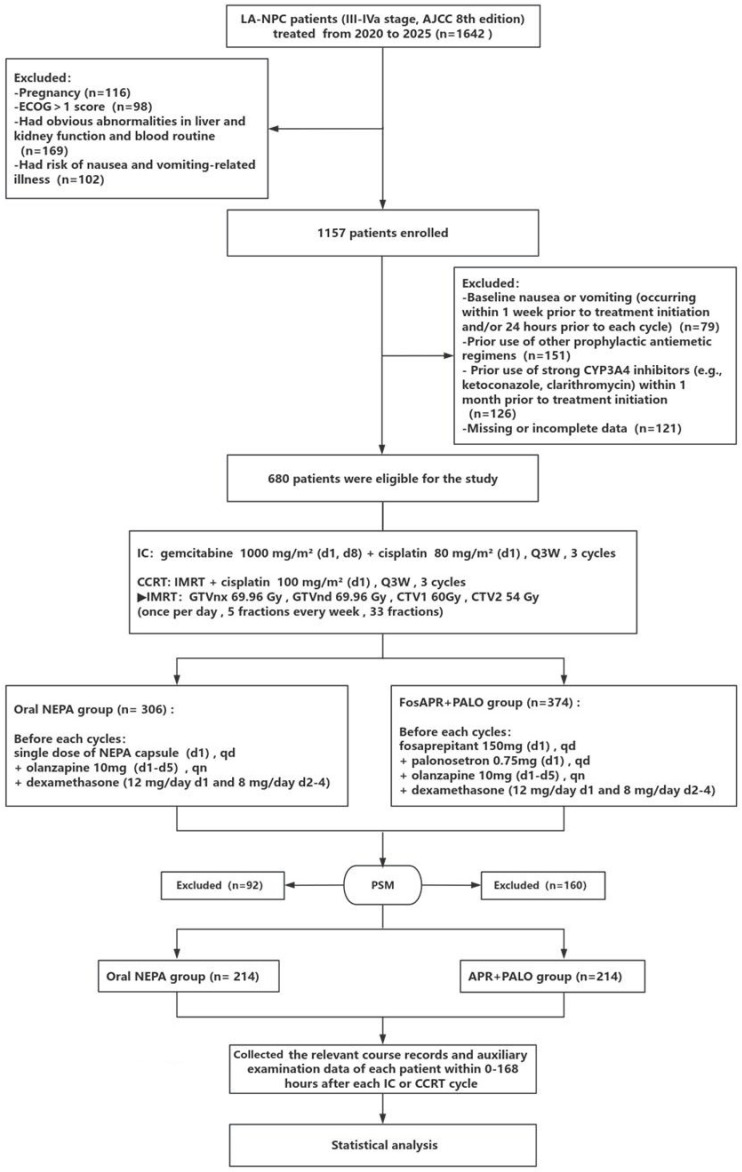
Study diagram. IMRT: Intensity-modulated radiation therapy, PSM: Propensity score matching.

Patients in the oral NEPA group received a single oral dose of NEPA capsules (Helsinn Birex Pharmaceuticals Ltd., Dublin, Ireland) one hour before the start of each chemotherapy cycle. Patients in the FosAPR (Jiangsu Hansoh Pharmaceutical Co., Ltd.) plus PALO (Jiangsu HaiCi Bio-Pharmaceutical Co., Ltd., Taizhou, China) group received an intravenous infusion of FosAPR 150 mg over 30 min, followed by a single intravenous injection of PALO 0.25 mg. Both groups received a single oral dose of dexamethasone (Tianjin SINE Jinjin Pharmaceutical Co., Ltd., Tianjin, China) 12 mg 30 min before chemotherapy, followed by dexamethasone 8 mg daily on days two through four. From day one to day four after chemotherapy, both groups received olanzapine (Jiangsu Hansoh Pharmaceutical Co., Ltd.) 5 mg orally once daily (Figure 1).

### 2.3. Assessments

#### 2.3.1. Data Collection and Outcome Definitions

Clinical data were extracted from the institutional electronic medical record system. Two investigators independently collected information using a standardized data abstraction form, with discrepancies resolved through discussion with a senior researcher. Data on vomiting episodes, retching, rescue antiemetic use, nausea severity, and adverse events were recorded for days 1 through 7 of each chemotherapy cycle based on documentation in medical records.

The primary endpoint was the CR rate during the extended overall phase (0–168 h) of the first IC cycle and the first CCRT cycle. CR was defined as the absence of emesis (vomiting or retching) and no use of rescue antiemetic medication. Other outcomes, including CR rates in other time phases, nausea control, time to treatment failure (TTF), and nutritional parameters, were designated as secondary or exploratory endpoints.

To account for the inherent differences between IC and CCRT in cisplatin dosing (80 mg/m^2^ vs. 100 mg/m^2^) and the addition of concurrent radiotherapy, efficacy outcomes were evaluated separately for each treatment phase. Stratified analyses by treatment phase were performed for all efficacy endpoints, with IC and CCRT analyzed as distinct clinical contexts. This approach allowed us to assess whether the treatment effect of oral NEPA differed between the two phases and to examine the consistency of findings across the overall treatment course.

#### 2.3.2. Nausea Severity and Adverse Events

Nausea severity was assessed daily using a 100 mm visual analog scale (VAS). As part of routine clinical care, nursing staff instructed patients to indicate their peak nausea intensity over the preceding 24 h, and the scores were transcribed into the medical records. All adverse events and CINV episodes were graded according to the Common Terminology Criteria for Adverse Events (CTCAE), version 5.0. Causality was evaluated using a five-point scale, with events classified as definite, probable, possible, unlikely, or unrelated considering treatment-related effects. Nausea and vomiting were treated as efficacy measures and were excluded from the safety analysis.

#### 2.3.3. Time Phases of Efficacy Assessment

Efficacy outcomes were evaluated across predefined time phases during the first IC and first CCRT cycles: acute (0–24 h), delayed (24–120 h), overall (0–120 h), extended delayed (24–168 h), beyond delayed (120–168 h), and extended overall (0–168 h). For subsequent treatment cycles, the analysis focused on the extended overall phase. For both IC and CCRT phases, the following endpoints were assessed separately: CR rates across all time phases, nausea control rates (no significant nausea and no nausea), and TTF. This phase-stratified assessment enabled us to determine whether the antiemetic efficacy of oral NEPA remained consistent across different treatment contexts or whether the effect varied by phase.

#### 2.3.4. Nutritional Status Evaluation

Nutritional status was evaluated based on changes in body weight, BMI, and Patient-Generated Subjective Global Assessment (PG-SGA) category. Trained dietitians performed PG-SGA assessments at baseline and within one week after completion of the third CCRT cycle. Patients were classified as category A (well-nourished), B (moderately malnourished), or C (severely malnourished) according to standard criteria [22,23,24]. Consistent with prior observations that CCRT has a more pronounced effect on body weight than IC in patients with NPC [24], the analysis prioritized weight changes during the CCRT phase. Percentage weight loss was calculated relative to baseline, with higher values indicating greater nutritional compromise.

### 2.4. Statistical Analysis

Statistical analyses were conducted using SPSS version 26.0 (IBM Corp., Armonk, NY, USA) and visualized with GraphPad Prism 9.0.0 (GraphPad Software, San Diego, CA, USA). Propensity score matching (PSM) with a 1:1 nearest neighbor algorithm and a caliper width of 0.02 was performed to balance baseline characteristics, including age, sex, pathology, BMI, TNM stage, and history of motion sickness. To minimize selection bias, we performed 1:1 PSM using a nearest neighbor algorithm with a caliper of 0.02, which produced 214 well balanced pairs. Post-matching balance was confirmed by standardized mean differences (SMDs) <0.1 across all covariates.

Descriptive statistics summarized demographic and clinical characteristics. Categorical variables were presented as frequencies and percentages, and continuous variables were assessed for normality using the Shapiro–Wilk test. Normally distributed data were reported as mean ± standard deviation (SD), whereas non-normally distributed data were expressed as median with interquartile range (IQR).

For the primary endpoint, differences in CR rates between the two groups were compared using the chi-square test. Logistic regression was used to estimate odds ratios (ORs) with 95% confidence intervals (CIs).

To evaluate whether the treatment effect differed between treatment phases, we performed stratified analyses by treatment phase (IC vs. CCRT) for the primary endpoint (CR rate during the extended overall phase of the first cycle). A logistic regression model including treatment group, treatment phase, and a treatment-by-phase interaction term was fitted to formally test for effect modification. A *p*-value for interaction <0.10 was considered indicative of potential heterogeneity.

For cycle-stratified analyses (cycles 1, 2, and 3 within IC and CCRT), generalized estimating equations (GEEs) with an exchangeable correlation structure were applied to account for within-patient correlation across repeated cycles. The model included treatment group, cycle number, and the treatment-by-cycle interaction term to assess whether the treatment effect varied over time.

Subgroup analyses based on prespecified clinical characteristics (sex, age, BMI, and history of motion sickness) were performed using logistic regression with interaction terms. All subgroup analyses were considered exploratory; therefore, no adjustment for multiple comparisons was applied.

TTF was estimated using the Kaplan–Meier method, with differences between groups compared using the log-rank test. Multivariate Cox proportional hazards regression provided adjusted hazard ratios (HRs) with 95% CIs.

Variables with more than 20% missing data were excluded from the analysis; remaining missing values were addressed using multiple imputation. All tests were two-sided, with statistical significance defined as *p* < 0.05, except for interaction tests where *p* < 0.10 was considered suggestive of heterogeneity.

## 3. Results

### 3.1. Patient Characteristics

**Figure 2:**
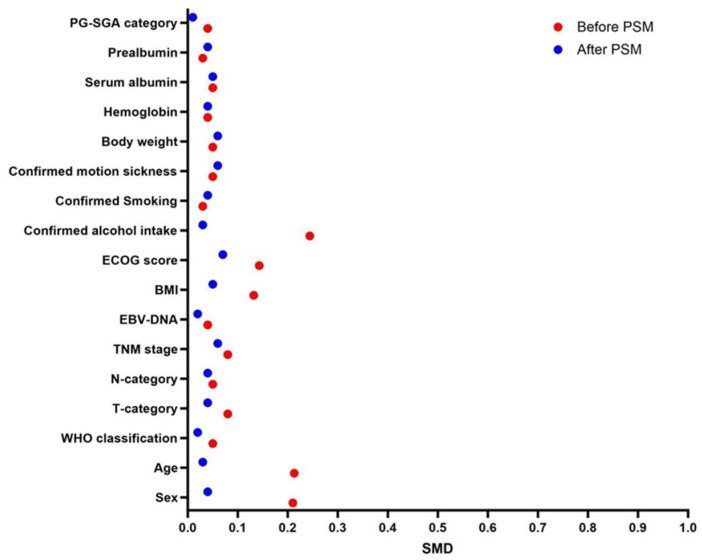
Covariate Balance Plot. Standardized mean differences (SMDs) before and after propensity score matching. All covariates had SMDs < 0.1 after matching, indicating good balance between groups.

A total of 1642 patients with LA-NPC were initially screened. After applying the inclusion and exclusion criteria, 306 patients were allocated to the oral NEPA group and 374 to the FosAPR + PALO group. Before matching, the two groups differed in sex, age, BMI, ECOG score, and alcohol intake; after matching, these characteristics were well balanced (Table 1). All SMDs were below 0.1 (Figure 2), indicating successful covariate balance.

### 3.2. Efficacy

The primary endpoint was the CR rate during the extended overall phase (0–168 h) of the first treatment cycle, assessed separately for IC and CCRT. During the first IC cycle, the oral NEPA group achieved a significantly higher CR rate than the FosAPR + PALO group in the extended overall phase (80.0% vs. 70.1%, *p* = 0.019). A similar difference was observed during the first CCRT cycle (74.8% vs. 64.5%, *p* = 0.021). No significant differences in CR rates emerged between the two groups in the acute phase. However, the oral NEPA group consistently showed numerically higher CR rates across the delayed, overall, extended delayed, and extended overall phases.

**Figure 3:**
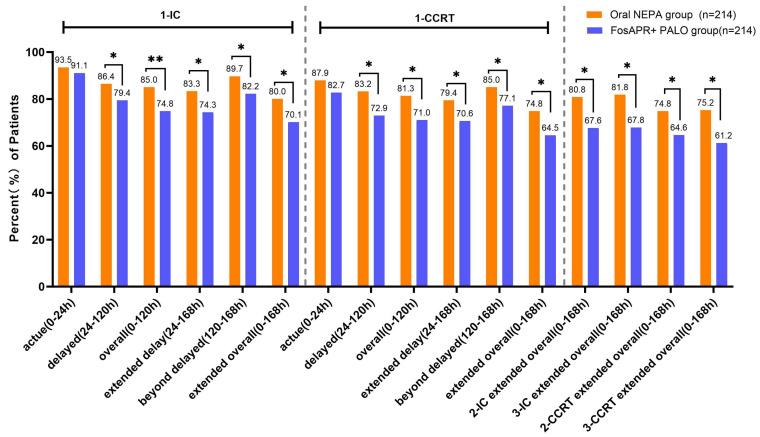
Complete response (CR) rates in the oral NEPA and FosAPR + PALO groups across predefined time phases after PSM. Data are percentages. 1-IC, first induction chemotherapy cycle; 1-CCRT, first concurrent chemoradiotherapy cycle; CR, complete response (no emesis and no rescue medication). * *p* < 0.05, ** *p* < 0.01 (chi-square test).

In subsequent treatment cycles, the CR benefit of oral NEPA was maintained during the extended overall phase. For the second and third IC cycles, CR rates were 80.8% vs. 67.6% (*p* = 0.002) and 81.8% vs. 67.8% (*p*= 0.001), respectively. During the second and third CCRT cycles, CR rates were 74.8% vs. 64.6% (*p* = 0.012) and 75.2% vs. 61.2% (*p* = 0.002), respectively, all favoring the oral NEPA group (Figure 3).

During the first IC cycle, the oral NEPA group showed higher rates of both no significant nausea and no nausea across all phases except the acute phase. For no significant nausea, the rates in the oral NEPA group compared with the FosAPR + PALO group were 85.6% versus 75.7% in the delayed phase (*p* = 0.010), 80.0% versus 71.0% in the overall phase (*p* = 0.033), 78.0% versus 68.7% in the extended delayed phase (*p* = 0.029), 88.3% versus 78.0% in the beyond delayed phase (*p* = 0.004), and 77.1% versus 65.9% in the extended overall phase (*p* = 0.010). Similar differences were observed for no nausea, with the oral NEPA group achieving statistically higher rates in the same phases (Table 2).

A comparable pattern emerged during the first CCRT cycle. The oral NEPA group consistently achieved higher rates of no significant nausea and no nausea across all phases except the acute phase, including the extended overall phase where the differences were 72.9% versus 60.3% (*p* = 0.006) for no significant nausea and 69.6% versus 56.5% (*p* = 0.005) for no nausea. In subsequent treatment cycles, the advantage of oral NEPA remained statistically significant during the CCRT phase, whereas consistency was less evident during the IC phase (Table 2).

**Figure 4:**
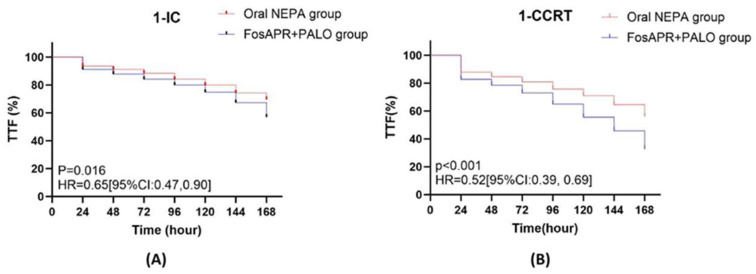
Time to treatment failure (TTF): first vomiting episode or rescue antiemetic use after PSM. Kaplan–Meier curves showing the cumulative incidence of treatment failure (first vomiting episode or rescue medication use) during the first IC (**A**) and first CCRT (**B**) cycles. Shaded areas represent 95% confidence intervals. *p*-values were calculated using the log-rank test.

TTF, defined as the time to first vomiting episode or use of rescue medication, is presented in Figure 4. The Kaplan–Meier curves began to separate after day 4 and remained divergent through day 7, with the oral NEPA group showing a more favorable TTF profile.

During the extended overall phase, the proportion of patients requiring rescue antiemetics was lower in the oral NEPA group than in the FosAPR + PALO group. In the first IC cycle, the rates were 14.5% versus 22.0% (*p* = 0.045), and in the first CCRT cycle, a favorable trend was observed (18.2% vs. 25.7%, *p* = 0.062). During IC and CCRT, the oral NEPA group had a longer median time to first rescue administration. In the first IC cycle, the mean time to first rescue was 58.32 ± 2.43 h for oral NEPA versus 51.32 ± 2.26 h for FosAPR + PALO, *p* < 0.001. In the first CCRT cycle, it was 58.27 ± 2.29 h for oral NEPA versus 52.19 ± 2.64 h for FosAPR + PALO, *p* < 0.001. The types of rescue agents used were similar between the two groups, with metoclopramide and prochlorperazine being the most common (Table 3).

### 3.3. Safety

Safety was assessed in all 428 matched patients. As summarized in Table 4, the antiemetic regimen based on oral NEPA was generally well tolerated, and the incidence of adverse events in both groups was similar. Constipation was the most frequently reported treatment-related adverse event (12.6% in the oral NEPA group vs. 14.0% in the FosAPR + PALO group). The incidence of grade ≥ 3 adverse events was 20.1% in the oral NEPA group and 21.5% in the APR + PALO group, with no statistically significant difference (*p* = 0.721). No serious adverse events were attributed to either antiemetic regimen.

### 3.4. Nutritional Status

**Figure 5:**
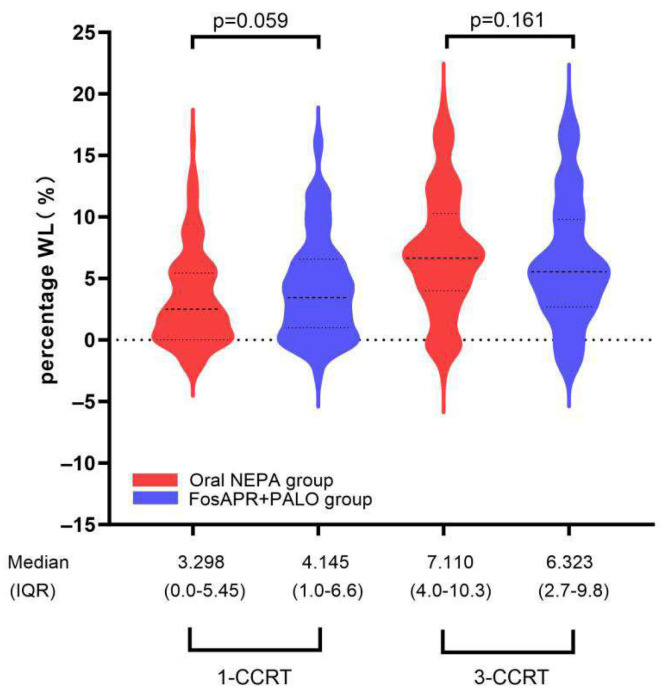
Longitudinal changes in body weight during concurrent chemoradiotherapy after PSM.

Changes in body weight during CCRT are shown in Figure 5. Weight loss increased progressively over the course of treatment, with no significant difference between groups at the end of the third CCRT cycle (oral NEPA vs. FosAPR + PALO, *p* = 0.161).

Ultimately, of 214 patients in the oral NEPA group, 68 (31.8%) showed weight loss ≥ 5.0%, whereas 80 (37.4%) in the FosAPR + PALO group showed weight loss ≥ 5.0%. We also compared patients who achieved WL ≥ 5.0% during the last CCRT and those who did not. Patients with cumulative WL ≥ 5.0% in the oral NEPA group were similar to those in the FosAPR + PALO group, with no statistically significant difference. Among patients who achieved WL ≥ 5.0%, patients with PG-SGA grade C of the oral NEPA and FosAPR + PALO groups were compared (41.2% vs. 43.8%, *p* = 0.948) (Table 5).

## 4. Discussion

Cisplatin-based chemotherapy remains the cornerstone of treatment for LA-NPC. Its high emetogenicity (emetic risk > 90% without prophylaxis) mandates rigorous supportive care [4]. The 2023 MASCC/ESMO guidelines recommend a triple regimen comprising an NK_1_RA, a 5-HT_3_RA, and dexamethasone for HEC, with olanzapine added for high-risk patients [7]. Despite clear guidance, real-world adherence remains suboptimal, largely due to the logistical complexity of multidrug regimens [8,9]. This study directly compared oral NEPA (a fixed-dose combination of netupitant and PALO) with intravenous FosAPR plus PALO in patients with LA-NPC undergoing multi-cycle cisplatin-based IC and CCRT. Our results indicate that oral NEPA provides superior and sustained CINV protection, particularly during the extended 0–168 h period, and that this advantage persists across repeated cycles.

In the extended overall phase, oral NEPA provided superior CINV control compared with the intravenous regimen, whereas acute phase protection was equivalent between the two groups. Efficacy divergence emerged after 24 h and persisted through day 7, a pattern consistent with the pharmacokinetic profiles of the two NK_1_RAs.

The temporal pattern of antiemetic efficacy observed in this study reflects the distinct pathophysiological phases of CINV. The acute phase (0–24 h) is primarily mediated by serotonin (5-HT) released from enterochromaffin cells of the small intestine following cisplatin-induced mucosal injury, which activates 5-HT_3_ receptors on vagal afferent nerves and the chemoreceptor trigger zone [12]. Both regimens provided comparable protection in this phase by blocking 5-HT_3_ receptors with palonosetron. In contrast, the delayed and extended phases (beyond 24 h) are predominantly driven by substance P, which is also released from enterochromaffin cells and acts on NK_1_ receptors in the nucleus tractus solitarius and the area postrema [12]. Netupitant, a highly selective NK_1_RA, maintains over 90% NK_1_ receptor occupancy at 96 h and generates active metabolites with prolonged half-lives, thereby providing sustained antagonism of the substance P pathway [17]. Conversely, fosaprepitant has a short terminal half-life of approximately 9–13 h [11], leaving the NK_1_ receptor inadequately blocked during the critical window when substance P signaling peaks. Importantly, in patients receiving concurrent chemoradiotherapy, radiation directly stimulates the dorsal vagal complex and other brainstem emetic centers, further amplifying substance P-mediated pathways and increasing overall emetic risk [20,21]. The superior extended-phase protection conferred by oral NEPA is therefore mechanistically consistent with its prolonged neurokinin-1 receptor occupancy.

Netupitant maintains >90% NK_1_ receptor occupancy at 96 h and generates active metabolites with prolonged half-lives, conferring durable substance P pathway antagonism [17]. In contrast, FosAPR has a relatively short terminal half-life, potentially leaving patients unprotected during the critical window for emetic memory consolidation [11,25].

This temporal pattern, initially overlapping efficacy followed by progressive separation, supports the hypothesis that extended receptor occupancy provides clinically meaningful protection beyond the conventional 120 h window. Consistent with this hypothesis, a recent meta-analysis of seven randomized controlled trials confirmed that the NEPA plus dexamethasone regimen achieved superior complete response rates compared with aprepitant-based regimens during both the overall phase (risk ratio = 1.15, 95% CI 1.06–1.25) and the delayed phase (risk ratio = 1.20, 95% CI 1.08–1.33), with no significant difference in adverse events [26].

The sustained efficacy of NEPA in subsequent treatment cycles is noteworthy. The CR benefit observed in the first cycle was maintained in subsequent cycles, whereas the comparator regimen showed diminished protection over time. These findings challenge the conventional view that antiemetic efficacy is cycle-independent. Robust first-cycle control may prevent anticipatory nausea and conditioned emetic responses, resetting the emetic threshold for later cycles [12]. The progressive separation of TTF curves after day 4 (Figure 4) provides visual evidence for this phenomenon. CR rates declined across cycles in the FosAPR + PALO group but remained stable or even improved in the NEPA group, suggesting that short-acting NK_1_RAs may fail to provide sufficient coverage when emetic responses become entrenched. Moreover, the recently published phase IV My Risk trial provided direct evidence that NEPA-based prophylaxis confers superior multi-cycle protection even in the moderately emetogenic chemotherapy (MEC) setting. In that study, patients with a high-risk CINV score who received NEPA plus dexamethasone achieved significantly higher complete response rates across three consecutive MEC cycles compared with standard 5-HT_3_RA plus dexamethasone (odds ratio 1.67, 95% CI 1.12–2.49, *p* = 0.012), with the NEPA group consistently showing better outcomes in no nausea, no emesis, and complete protection [27]. Although our study focused on HEC with cisplatin, the My Risk findings reinforce the principle that a fixed-dose NK_1_RA/5-HT_3_RA combination offers durable, cycle-persistent benefits, particularly when patient-specific risk factors are considered. These data extend the CONSOLE study finding that fosnetupitant outperformed FosAPR during the 120 to 168 h window by demonstrating sustained multi-cycle benefits in a real-world population receiving combined-modality therapy [18].

The study also revealed marked differences in emetic risk between treatment phases. Despite identical antiemetic prophylaxis, patients experienced higher CINV rates during CCRT than during IC. Two factors likely explain this. First, the cisplatin dose was higher during CCRT (100 mg/m^2^) than IC (80 mg/m^2^), and cisplatin emetogenicity is dose-dependent [4]. Second, IMRT delivers substantial radiation to the brainstem, particularly the dorsal vagal complex, a key emetic center [20,21]. High-dose cisplatin combined with brainstem irradiation likely produces additive or synergistic emetic stimuli, placing greater demands on antiemetic coverage. Notably, NEPA maintained its advantage over FosAPR + PALO even during this higher-risk CCRT phase, reinforcing the value of prolonged receptor occupancy in challenging clinical settings.

Nausea control, often more distressing and harder to manage than vomiting, similarly favored NEPA. The NEPA group achieved significantly higher rates of no significant nausea and no nausea during the extended overall phase of both IC and CCRT, with statistically significant differences in the delayed and beyond delayed phases. These findings are clinically meaningful, as persistent nausea can impair oral intake, reduce quality of life, and drive treatment non adherence independent of vomiting. This observation is corroborated by a recent prospective study by Katta and colleagues, which found that chemotherapy-induced nausea and vomiting (CINV) remain among the most distressing patient-reported side effects in routine clinical practice, directly compromising patients’ emotional well-being, quality of life, and adherence to treatment [28]. The high incidence of patient-reported vomiting (44.2%) and loss of appetite in that study underscores the substantial burden of suboptimal CINV control, particularly when nausea persists beyond the acute phase. The consistent superiority of NEPA in controlling nausea across multiple cycles suggests that the fixed-dose combination may offer particular benefits during the delayed and extended phases, where substance P-mediated signaling predominates [12].

Despite clear advantages in CINV control, NEPA did not translate into improved nutritional outcomes at treatment completion. This finding, though initially unexpected, highlights a critical competing risk phenomenon in patients undergoing CCRT for LA-NPC. Radiation-induced oral mucositis (RIOM) affected over 70% of patients in both groups (Table 4), directly impairing oral intake and overshadowing any potential nutritional benefit from improved CINV control [22,29]. The absence of intergroup differences in nutritional outcomes suggests that mucositis, rather than CINV, is the dominant driver of weight loss and malnutrition during CCRT. This observation carries important clinical implications. Optimal antiemetic prophylaxis, while essential for patient comfort and treatment adherence, is necessary but not sufficient to prevent nutritional decline. Comprehensive supportive care must concurrently address mucositis through preventive measures, aggressive pain management, and timely nutritional support, including enteral feeding when indicated.

The safety profiles of the two antiemetic regimens were comparable. Constipation, a predictable consequence of 5HT3 receptor antagonism from reduced gastrointestinal motility, was the most frequently reported treatment-related adverse event, occurring in 12.8% of the NEPA group and 14.1% of the FosAPR + PALO group. The incidence of grade ≥ 3 adverse events was 20.1% versus 21.5%, with no significant difference, and no serious adverse events were attributed to either regimen. These findings indicate that the enhanced efficacy of NEPA was not achieved at the expense of increased toxicity. Consistent with this, Marshall and colleagues reported that CINV-related dietary restriction affects 10% of chemotherapy patients and often goes unaddressed, contributing to weight loss [30]. The ESPEN guidelines therefore advocate for routine nutritional assessment and timely enteral support in high-risk populations such as patients receiving CCRT [31].

The practical advantages of NEPA deserve consideration. Its single-capsule, once-per-cycle oral regimen simplifies prophylaxis compared to the multi-step intravenous regimen of FosAPR followed by PALO. However, RIOM in patients with LA-NPC receiving concurrent cisplatin can cause dysphagia, potentially hindering oral intake. In this study, the oral capsule was administered before mucositis became severe, and importantly, an intravenous formulation of NEPA is available with proven non-inferior efficacy to the oral capsule [32]. This offers a clear advantage that patients who develop significant dysphagia can switch to IV NEPA without compromising antiemetic protection, whereas the FosAPR + PALO regimen lacks such a fixed-dose alternative. Thus, even though our study used the oral formulation (as the IV form was not yet available at our institution), the overall evidence supports NEPA as a flexible and effective option across the full course of CCRT.

Several limitations should be noted. The single-center retrospective design may introduce selection bias despite propensity score matching, and residual confounding from unmeasured variables (e.g., undocumented comorbidities, psychosocial factors) cannot be ruled out. The exclusively Chinese cohort limits generalizability to other populations. Additionally, patient-reported nausea severity was derived from routine clinical documentation rather than prospective standardized collection, which may introduce variability.

Future multicenter prospective studies with uniform data collection standards are needed to validate these findings. Such studies should incorporate formal quality-of-life assessments using validated instruments, systematic documentation of nutritional support, and detailed recording of mucositis severity to better understand the competing risks shaping nutritional outcomes. Randomized controlled trials comparing NEPA with other NK_1_RA-based regimens would provide the highest level of evidence. Cost effectiveness analyses comparing NEPA with multi-step intravenous regimens would inform clinical practice guidelines and reimbursement decisions. Finally, research exploring the optimal integration of antiemetic therapy with nutritional interventions and mucositis management could identify strategies to improve both symptom control and nutritional outcomes in this vulnerable population.

## 5. Conclusions

This study suggests that oral NEPA may provide durable CINV prophylaxis with a favorable safety profile in patients with NPC undergoing IC and CCRT. While optimized CINV control is expected to improve nutritional intake, the comparable intergroup nutritional outcomes (weight loss ≥ 5% and severe malnutrition rates) suggest that radiotherapy-induced toxicities predominantly drive nutritional deterioration. By replacing complex multidrug regimens with a single capsule, oral NEPA enhances guideline adherence and optimizes the benefit–risk profile. However, prospective randomized controlled trials are needed to confirm these findings.

## Figures and Tables

**Table 1:** Baseline demographics and clinical characteristics before and after propensity score matching (PSM).

Characteristics	Before PSM			After PSM		
	Oral NEPA (*n* = 306)	FosAPR + PALO (*n* = 374)	*p*	Oral NEPA (*n* = 214)	FosAPR + PALO (*n* = 214)	*p*
Sex, *n* (%)			0.006			0.771
Female	143 (46.7)	214 (57.2)		101 (47.2)	98 (45.8)	
Male	163 (53.3)	160 (42.8)		113 (52.8)	116 (54.2)	
Age, *n* (%)			0.005			0.765
<45 years	93 (30.4)	153 (40.9)		82 (38.3)	79 (36.9)	
≥45 years	213 (69.6)	221 (59.1)		132 (61.7)	135 (63.1)	
WHO classification, *n* (%)			0.560			0.789
Keratinizing, undifferentiated	52 (17.0)	70 (18.7)		34 (15.9)	32 (15.0)	
Non-keratinizing, undifferentiated	254 (83.0)	304 (81.3)		180 (84.1)	182 (85.0)	
T-category, 8th, *n* (%)			0.164			0.622
T1–T2	101 (33.0)	105 (28.1)		84 (39.3)	89 (41.6)	
T3–T4	205 (67.0)	269 (71.9)		130 (60.7)	125 (58.4)	
N-category, 8th, *n* (%)			0.450			0.670
N0–1	78 (25.5)	105 (28.1)		60 (28.0)	64 (29.9)	
N2–3	228 (74.5)	269 (71.9)		154 (72.0)	150 (70.1)	
TNM stage, 8th, *n* (%)			0.258			0.438
III	117 (38.2)	159 (42.5)		95 (44.4)	103 (48.1)	
IVa	189 (61.8)	215 (57.5)		119 (55.6)	111 (51.9)	
EBV-DNA, *n* (%)			0.450			0.846
<500	127 (41.5)	166 (44.4)		100 (46.7)	102 (47.7)	
≥500	179 (58.5)	208 (55.6)		114 (53.3)	112 (52.3)	
BMI, *n* (%)			0.026			0.560
<22 kg/m^2^	114 (37.2)	171 (45.7)		94 (43.9)	100 (46.7)	
≥22 kg/m^2^	192 (62.7)	203 (54.3)		120 (56.1)	114 (53.3)	
ECOG score, *n* (%)			0.025			0.457
0	218 (71.2)	236 (63.1)		155 (72.4)	148 (69.1)	
1	88 (28.8)	138 (36.9)		59 (27.6)	66 (30.8)	
Confirmed alcohol intake, *n* (%)	94 (30.7)	71 (19.0)	0.001	40 (18.7)	37 (17.3)	0.706
Confirmed smoking, *n* (%)	212 (69.3)	254 (67.9)	0.703	154 (72.0)	150 (70.1)	0.670
Confirmed motion sickness, *n* (%)	43 (14.1)	45 (12.0)	0.435	23 (10.7)	19 (9.0)	0.516
Body weight, kg, mean ± SD	62.53 ± 4.76	63.75 ± 3.87	0.556	61.23 ± 3.64	62.54 ± 3.32	0.432
Hemoglobin, g/L, mean ± SD	128.5 ± 5.86	127.3 ± 6.64	0.743	127.2 ± 3.64	128.1 ± 3.54	0.689
Serum albumin, g/L, mean ± SD	41.23 ± 3.42	42.76 ± 3.46	0.564	42.54 ± 2.76	43.64 ± 2.76	0.587
Prealbumin, mg/L, mean ± SD	234.34 ± 10.21	232.12 ± 9.21	0.834	228.84 ± 8.22	226.76 ± 8.37	0.743
PG-SGA category, *n* (%)			0.755			0.986
A	225 (73.5)	271 (72.5)		154 (71.9)	148 (69.2)	
B	81 (26.5)	103 (27.5)		60 (28.0)	66 (30.8)	
C	0	0		0	0	

Data are presented as *n* (%) or mean ± SD. *p*-values were calculated using the chi-square test for categorical variables and the independent *t*-test for continuous variables. BMI, body mass index; ECOG, Eastern Cooperative Oncology Group; PG-SGA, Patient-Generated Subjective Global Assessment. Confirmed alcohol intake: ≥150 mL of alcohol per week for six consecutive months before diagnosis. Confirmed smoking: cumulative lifetime smoking history of ≥100 cigarettes, with continued smoking at diagnosis or within six months before diagnosis. Confirmed motion sickness: self-reported history documented during baseline medical interviews. PG-SGA categories: A, well-nourished; B, moderately malnourished; C, severely malnourished.

**Table 2:** Nausea control in the oral NEPA and FosAPR + PALO groups across predefined time phases After PSM.

Cycle	Phase	No Significant Nausea (VAS < 25 mm), *n* (%)			No Nausea (VAS = 0 mm), *n* (%)		
		Oral NEPA (*n* = 214)	FosAPR + PALO (*n* = 214)	*p*	Oral NEPA (*n* = 214)	FosAPR + PALO (*n* = 214)	*p*
1-IC	Acute	193 (90.2)	187 (87.4)	0.358	180 (84.1)	165 (77.1)	0.067
	Delayed	183 (85.6)	162 (75.7)	**0.010**	166 (77.6)	142 (66.4)	**0.010**
	Overall	171 (80.0)	152 (71.0)	**0.033**	162 (75.6)	140 (65.4)	**0.020**
	Extended delayed	167 (78.0)	147 (68.7)	**0.029**	158 (73.8)	136 (63.6)	**0.022**
	Beyond delayed	189 (88.3)	167 (78.0)	**0.004**	169 (79.0)	142 (66.4)	**0.003**
	Extended overall	165 (77.1)	141 (65.9)	**0.010**	154 (72.0)	130 (60.7)	**0.014**
1-CCRT	Acute	171 (80.0)	160 (74.8)	0.204	163 (76.2)	142 (66.4)	0.165
	Delayed	162 (75.7)	138 (64.5)	**0.011**	151 (70.6)	130 (60.0)	**0.033**
	Overall	155 (72.4)	134 (62.6)	**0.030**	147 (68.7)	127 (59.3)	**0.044**
	Extended delayed	152 (71.0)	129 (60.3)	**0.019**	154 (72.0)	127 (59.3)	**0.006**
	Beyond delayed	166 (77.6)	137 (64.0)	**0.002**	156 (72.9)	132 (61.7)	**0.013**
	Extended overall	156 (72.9)	129 (60.3)	**0.006**	149 (69.6)	121 (56.5)	**0.005**
2-IC	Extended overall	162 (75.7)	132 (61.7)	**0.002**	150 (70.1)	125 (58.4)	**0.012**
3-IC	Extended overall	158 (73.8)	131 (61.2)	**0.005**	145 (67.8)	122 (57.0)	**0.022**
2-CCRT	Extended overall	150 (70.1)	126 (59.0)	**0.015**	140 (65.4)	118 (55.1)	**0.030**
3-CCRT	Extended overall	148 (69.2)	128 (59.8)	**0.043**	141 (65.9)	116 (54.2)	**0.014**

Data are presented as *n* (%). IC, induction chemotherapy; CCRT, concurrent chemoradiotherapy; VAS, visual analog scale. No significant nausea: VAS score < 25 mm; no nausea: VAS score = 0 mm. *p*-values were calculated using the chi-square test. Bold values indicate statistically significant differences (*p* < 0.05).

**Table 3:** Rescue antiemetic use during the extended overall phase (0–168 h).

Parameter	1-IC			1-CCRT		
	Oral NEPA (*n* = 214)	FosAPR + PALO (*n* = 214)	*p*	Oral NEPA (*n* = 214)	FosAPR + PALO (*n* = 214)	*p*
Patients requiring rescue, *n* (%)	31 (14.5)	47 (22.0)	0.045	39 (18.2)	55 (25.7)	0.062
Time to first rescue, hours, median (mean ± SD)	58.32 ± 2.43	51.32 ± 2.26	<0.001	58.27 ± 2.29	52.19 ± 2.64	<0.001
Type of rescue medication, *n* (%)			0.918			0.852
Metoclopramide	14 (45.2)	23 (48.9)		18 (46.2)	27 (49.1)	
Prochlorperazine	9 (29.0)	15 (31.9)		11 (28.2)	18 (32.7)	
Dolasetron	6 (19.4)	7 (14.9)		8 (20.5)	8 (14.5)	
Other	2 (6.5)	2 (4.3)		2 (5.1)	2 (3.6)	

Data are presented as *n* (%), median (IQR), or mean ± SD. 1-IC, first induction chemotherapy cycle; 1-CCRT, first concurrent chemoradiotherapy cycle. *p*-values were calculated using the chi-square test for proportions, the log-rank test for time-to-event, and the Mann–Whitney U test for number of doses.

**Table 4:** Summary of adverse events in patients who completed the entire study After PSM.

Adverse Reactions	Grade 1–2, *n* (%)		Grade 3–4, *n* (%)		*p*
	Oral NEPA (*n* = 214)	FosAPR + PALO (*n* = 214)	Oral NEPA (*n* = 214)	FosAPR + PALO (*n* = 214)	
Leukopenia	41 (19.2)	55 (25.7)	21 (9.8)	23 (10.7)	0.221
Anemia	57 (26.6)	59 (27.6)	12 (5.6)	14 (6.5)	0.885
Thrombocytopenia	30 (14.0)	38 (17.8)	18 (8.4)	14 (6.5)	0.475
AST increase	30 (14.0)	34 (16.0)	12 (5.6)	14 (6.5)	0.775
ALT increase	29 (13.6)	31 (14.5)	18 (8.4)	18 (8.4)	0.961
Creatinine increase	38 (17.8)	41 (19.2)	14 (6.5)	19 (9.0)	0.584
Constipation	27 (12.6)	30 (14.0)	0	0	0.670
Diarrhea	3 (1.4)	6 (2.8)	0	0	0.312
Dyspepsia	19 (9.0)	25 (11.7)	0	0	0.340
Cough	6 (2.8)	8 (3.7)	0	0	0.587
Infections	23 (10.8)	30 (14.0)	0	0	0.382
Headache	12 (5.6)	14 (6.5)	0	0	0.686
Fatigue	6 (2.8)	8 (3.7)	0	0	0.587
Mucositis oral cavity	158 (73.8)	166 (77.6)	40 (18.6)	42 (19.6)	0.395
Alopecia	134 (62.6)	137 (64.0)	6 (2.8)	6 (2.8)	0.953

Data are presented as *n* (%). Adverse events were graded according to CTCAE version 5.0. *p*-values were calculated using the chi-square test or Fisher’s exact test, comparing overall incidence (all grades) between groups. AST, aspartate aminotransferase; ALT, alanine aminotransferase.

**Table 5:** Comparison of 3-CCRT for cumulative WL5.0 (WL ≥ 5.0%) after PSM.

PG-SGA, *n* (%)	WL ≥ 5.0%		*p*	WL < 5.0%		*p*
	Oral NEPA (*n* = 68)	FosAPR + PALO (*n* = 80)		Oral NEPA (*n* = 146)	FosAPR + PALO (*n* = 134)	
A	8 (11.8)	10 (12.5)	0.948	88 (60.3)	79 (59.0)	0.974
B	32 (47.1)	35 (43.8)		43 (29.5)	41 (30.6)	
C	28 (41.2)	35 (43.8)		15 (10.3)	14 (10.4)	

Data are presented as *n* (%). 3-CCRT, third concurrent chemoradiotherapy cycle; PG-SGA, Patient-Generated Subjective Global Assessment; WL, weight loss. *p*-values were calculated using the chi-square test.

## Data Availability

The data that support the findings of this study are available from the corresponding author upon reasonable request.

## Abbreviations

The following abbreviations are used in this manuscript:

5-HT_3_RA	5-hydroxytryptamine-3 receptor antagonist
AJCC	American Joint Committee on Cancer
ALT	alanine aminotransferase
ASCO	American Society of Clinical Oncology
AST	aspartate aminotransferase
BMI	body mass index
CCRT	concurrent chemoradiotherapy
CI	confidence interval
CINV	chemotherapy-induced nausea and vomiting
CR	complete response
CSCO	Chinese Society of Clinical Oncology
CTCAE	Common Terminology Criteria for Adverse Events
DNA	deoxyribonucleic acid
EBV	Epstein–Barr virus
ECOG	Eastern Cooperative Oncology Group
ESMO	European Society for Medical Oncology
FosAPR	fosaprepitant
GEE	generalized estimating equations
HEC	highly emetogenic chemotherapy
HR	hazard ratio
IC	induction chemotherapy
IMRT	intensity-modulated radiation therapy
IQR	interquartile range
LA-NPC	locally advanced nasopharyngeal carcinoma
MASCC	Multinational Association of Supportive Care in Cancer
MAT	MASCC Antiemesis Tool
MEC	moderately emetogenic chemotherapy
NEPA	fixed-dose combination of netupitant and palonosetron
NK_1_RA	neurokinin-1 receptor antagonist
NPC	nasopharyngeal carcinoma
OR	odds ratio
PALO	palonosetron
PG-SGA	Patient-Generated Subjective Global Assessment
PSM	propensity score matching
RIOM	radiation-induced oral mucositis
SD	standard deviation
SMD	standardized mean difference
TTF	time to treatment failure
ULN	upper limit of normal
VAS	visual analog scale
WHO	World Health Organization
